# A systematic review of perinatal palliative care models: challenges and opportunities for the future

**DOI:** 10.1007/s00431-025-06459-0

**Published:** 2025-10-12

**Authors:** Anna Zanin, Annalisa Salerno, Maria Elena Cavicchiolo, Chiara Daicampi, Beatrice Martini, Anna Marinetto, Sabrina Salvadori, Franca Benini

**Affiliations:** 1 Women, Children and Adolescent Department, HUG Children’s Hospital, Hopitaux Universitaires de Genève, Geneva, Switzerland; 2 Palliative Care and Pain Service, Department of Women’s and Children’s Health, University of Padua, Padua, Italy; 3 Neonatal Intensive Care Unit, Department of Women’s and Children’s Health, University of Padua, Padua, Italy; 4 Department of Medicine, University of Padua, Padua, Italy; 5 Department of Health Professions, University Hospital of Padua, Padua, Italy

**Keywords:** Neonatal palliative care, Perinatal palliative care, Newborn, Neonate, Life-limiting condition, Life-threatening condition

## Abstract

Perinatal palliative care (PnPC) is critical for improving the quality of life of infants with extreme prematurity and life-limiting conditions. However, its implementation differs significantly worldwide. This systematic review will assess and compare PnPC models in neonatal intensive care units (NICUs) to identify best practices, challenges, and areas for improvement. Following PRISMA guidelines, a systematic search of PubMed, Embase, and CINAHL was conducted for studies published in the last decade describing PnPC delivery models. Data on study design, PnPC model characteristics, team composition, timing of involvement, and key outcomes were extracted and synthesized. Fourteen observational studies were included; no interventional trials were identified. The majority of studies originated from the USA (64%). Three primary PnPC delivery models were identified: consultative, integrative, and team-collaborative. Consultative models improved referral rates but were often associated with delays, whereas integrative and collaborative models provided more seamless, continuous care. Across all models, multidisciplinary teams were central to care delivery. Early integration of PnPC, particularly from the antenatal period, was associated with improved outcomes, including higher parental satisfaction, clearer goals of care, and reduced aggressive end-of-life interventions.

*Conclusion*: Early, integrated PnPC delivered by a multidisciplinary team improves end-of-life care and family support in the NICU. However, significant heterogeneity in practice and a lack of high-level evidence persists. Future research should prioritize rigorous interventional designs, greater geographical diversity, and the use of standardized, family-centered outcome measures to establish evidence-based guidelines for neonatal palliative care.

**Table:** 

**What is known: ** • *Perinatal Palliative Care is a vital part of neonatology, prioritizing the quality of life for extremely preterm infants and those with life-limiting conditions.*
**What is New:** • *The review revealed variability in perinatal palliative care models, timing and team compositions, suggesting early integration can improve support for babies, families and healthcare teams.* • *Future research should focus on rigorous interventional designs, geographic diversity, and long-term family centered outcomes.* • *Integrating practical clinical experience with theoretical education strengthens healthcare professionals’ competencies and guidelines development.*

**Supplementary Information:**

The online version contains supplementary material available at 10.1007/s00431-025-06459-0.

## Introduction

Neonatology emerged as a distinct field within pediatrics in the early twentieth century, evolving from providing basic supportive care [1] to an advanced specialty capable of actively providing “neonatal intensive care” saving extremely premature infants and surgically correcting once-fatal anomalies [2]. The introduction of exogenous surfactant therapy together with the introduction of many other innovations was a game-changer for preterm infants [3–9]; however, this increased survival often comes with severe complications affecting both the infant and their family [10, 11].

In response, perinatal palliative care (PnPC) emerged in the 1990 s to prioritize quality of life [2, 12–14]. Despite its recognized importance by the American Academy of Paediatrics and the World Health Organization international guidelines [15, 16], PnPC is not implemented consistently in the neonatal intensive care units (NICUs) worldwide due to resource and organizational hurdles [17]. Therefore, this systematic review aims to evaluate and compare PnPC models in NICUs to identify best practices and challenges in end-of-life (EoL) and supportive care.

## Materials and methods

### Search strategy

This systematic review adhered to PRISMA guidelines [18] and was registered with PROSPERO (CRD420250651816), not requiring IRB approval. In collaboration with a Librarian, we searched PubMed, Embase, and CINAHL for studies on palliative care models in NICUs published in the last 10 years, up to January 13, 2025 (see Appendix 1 for full search strategies).

### Inclusion and exclusion criteria

Eligible studies (randomized controlled trials, observational studies, surveys) described a model of perinatal palliative care delivery. We excluded letters, editorials, seminar reviews, small case series (< 5 patients), and non-English, Italian, or French articles.

### Data management, study selection, and data extraction

Using Covidence® (Veritas Health Innovation, Level 10, 446 Collins St, Melbourne VIC 3000, Australia), three authors independently (AM, AS, BM) screened titles and abstracts, with two authors required for an article to proceed to full-text review. Two independent reviewers among all the authors assessed the full texts, with a senior author (AZ) resolving any discrepancies. Reference lists of included articles were also screened for additional studies. Information collected included: study characteristics (e.g., first author’s name, publication year, journal, country, duration), study design, model of PnPC delivery, PnPC team composition, indications, and timing of palliative care involvement, quality improvement elements, and relevant outcomes associated with the described model. Quality Appraisal and Risk of Bias of the included studies was also assessed.

## Results

**Fig. 1:**
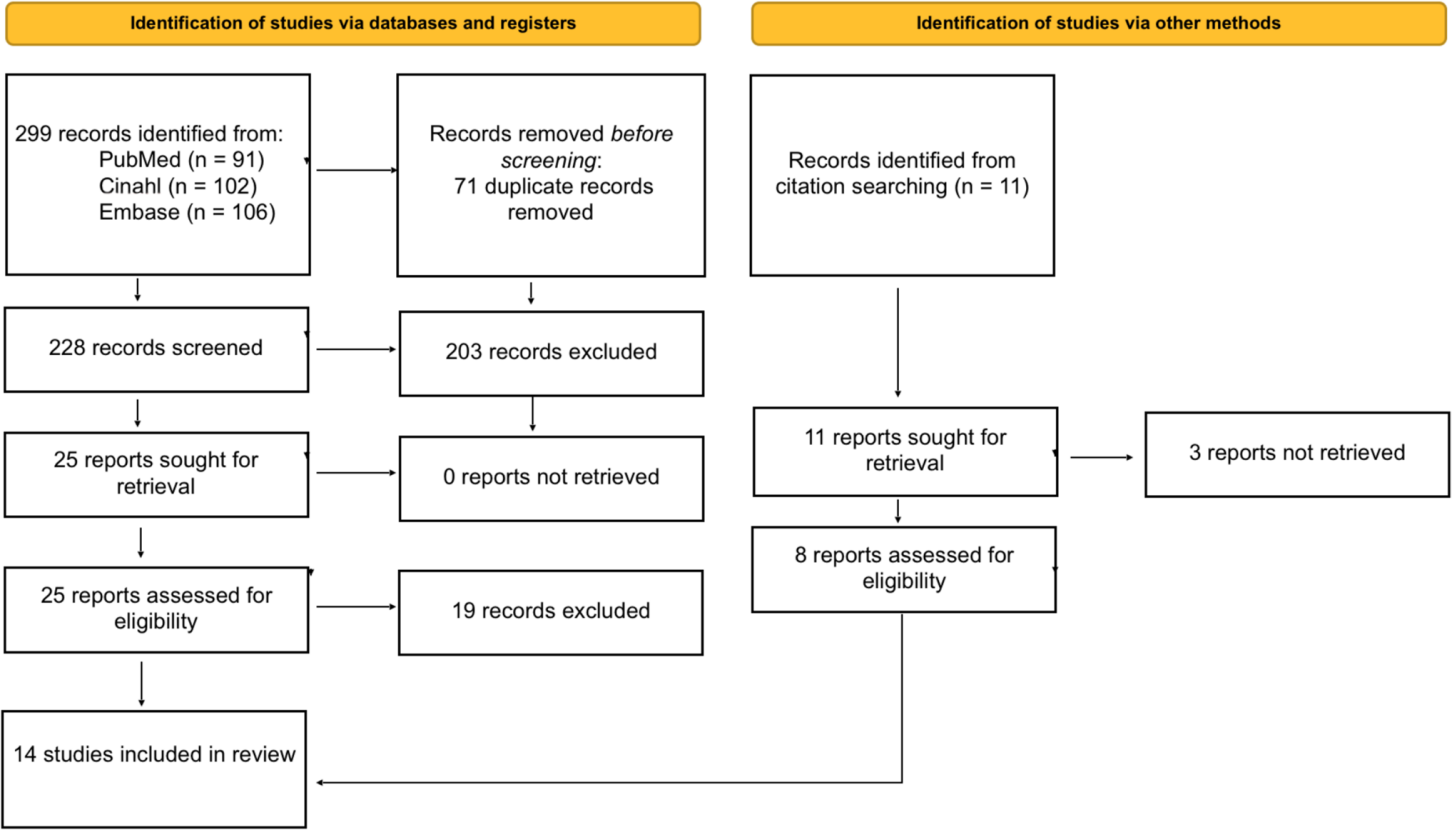
PRISMA 2020 flow diagram (according to Page MJ et al.) outlining the research strategy

Our initial search yielded 299 records. After removing 71 duplicates, we screened 228 abstracts, which led to 25 articles for full-text review. An additional 11 studies were identified from reference Lists. Of these 36 articles, 22 were excluded, resulting in 14 studies being included in the final review. The complete selection process is outlined in the PRISMA flow diagram (Fig. 1).

### Characteristics of the included studies

Among the selected studies, no interventional trials were identified. Four studies employed a retrospective design [19–22], while two were prospective in nature [23, 24]. Four studies adopted a mixed-methods approach [25–28], two were descriptive or prospective articles [29, 30], one was an observational time-series [30], and one a cross-sectional survey [31]. Eight studies (57%) were published in the last 5 years [19–23, 25, 29, 31]. Most of the studies were conducted in the USA (*n* = 9, 64%) [19, 21, 22, 24, 26–28, 30, 32], followed by Europe (*n* = 4; two in Italy and two in the UK) [20, 23, 29, 31], and one in Singapore [25]. Two studies were multicentric [21, 31].

The mainline characteristics of the included studies are summarized in Table 1.

**Table 1:** Summary of principle findings of reviewed studies (DNR: do not resiscitate order, WLS: Withholding and Withdrawal of Life Support, NICU: neonatal intensive care unit, PPC: pediatric palliative care, PnPC: perinatal palliative care, EoL: end of life)

Author and journal	Study design	Country	Unit and population (*n*)	Timing (months)	PnPC delivery model	PC team	Reason for PnPC involvement*	Timing of PnPC involvement	Quality improvement (yes/no)	Outcome evaluated	Outcome reached	Limits
Tewani et al., 2022 (Journal of Palliative Care)	Mix method study	Singapore	Regional referral center with 40-bed NICU and 60-bed high dependency unit (41)	48 months (January 2017-December 2019)	Consultative model (PeriPal service), role of coordination	PPC specialist, obstetrician, neonatologists, nurses and medical social workers	To provide individually tailored holistic care for expectant and new parents with a diagnosis of life-limiting fetal condition	From prenatal to postnatal referrals	Yes, positive feedback from families	N/A	N/A	-No outcomes definitions -- No measurement tools for PnPC impact, -small sample, -short follow up time
Samsel C et al., 2015 (Journal of Perinatology)	Retrospective and prospective chart review study	USA	80-bed regional level IV NICU(109)	64 months: 24 pre + 16 months during intervention implementation + 24 months after the implementation (January 2008–April 2013)	From a consultative model to a team collaborative model (not available a separate PPC consultative team)	Attending physicians, fellows, residents, nurse practitioners, nurses, nurse managers, respiratory therapists, occupational therapists, lactation consultants, pharmacists, nutritionists social workers, chaplains bereaved family members	EoL	From prenatal discussion meeting om 2 months basis	Yes	In the last 48 h of life, the decision to implement redirection of care, outcome expectation meetings between providers and families, the use of palliative medications and the presence of ancillary service consultation between the three epochs	Yes, significantly increased redirection of care (35% pre vs 73% post) and palliative medication use in the last 48 h of life (36% pre vs 64% post) Outcomes expectation meetings between providers and families and ancillary service consultation increased but data did not show statistical significance	Retrospective nature, ethical considerations regarding intervention withholding, the feasibility of an ideal washout period, the recognition of the variability in EoL care needs, potential data Limitations associated with chart reviews and outcome measure assessed restricted to the final 48 h of life
Younge et al., 2015 (J Prenatal)	Retrospective and prospective chart review study	USA	67-bed regional level IV NICU (150)	84 months: 36 pre-implementations + 48 (not included in the analysis) + 48 post-implementation (January 2003–December 2009)	Collaborative model	Neonatologists, advanced practice nurses, bedside nurses, social workers, pharmacologists, and chaplains, and included a neonatologist who is board-certified in Hospice and Palliative Medicine	EoL	From prenatal discussion	Yes	Impact of Neonatal Palliative Care Program on EoL care (evaluated as differences in demographics, cause of and age at death, family meetings, morphine dosage, use of benzodiazepines, use of neuromuscular blockers, DNR ) orders and WLS)	The program did not significantly change the incidence of WLS or overall mortality in the NICU. Benzodiazepine use increased in the post-implementation Era (26% vs. 43%, *p* = 0.03). Family meetings to discuss end-of-life care and DNR orders occurred more frequently in the subgroup of infants with activated palliative care order sets	Retrospective Nature, Limited Control Group (possible confounders by other factors that changed during that period), focus on a few specific outcomes (WLS, DNR orders, medication use) and may not have captured the full impact of the palliative care program on other important aspects
Tucker H. et al., 2020 (J Perinatology)	Retrospective chart review	USA	IV level neonatal intensive care unit	96 months (2010–2018)	Consultative model	PPC specialist	From prenatal discussion to postnatal referrals upon request	From prenatal discussion to pediatric follow up	No	Perceived values added for patients and neonatal team (subjective). Number of consultations per year; duration of follow-up of children evaluated	The use of PnPC increased over time. Added value for neonatologists: continuity with longitudinal support for the patients and a partnership with the neonatal team	Absence of valid quality indicators Number of consultations per years as indirect measures of PnPC effectiveness No parent and provider satisfaction detailed
Locatelli et al., 2020 (Frontiers)	Perspective article	Italy	III level NICU (n=300/year)	84 months (2013–2020)	Integrative and consultative model + specialist available for consultation	Obstetricians and neonatologists, midwives Professionals from other specialties available for consultation	Facilitate a care plan definition for neonates with a prenatal or postnatal diagnosis of life-limiting condition	From prenatal counseling to postnatal care bridge to PPC follow up	Yes	Number of pregnancies and outcome; neonatal outcomes	N/A	No clear evaluation of PCC program development; positive experience of parents of children in care that cannot be quantified by objective measures
Bolognani M et al., 2021 (Frontiers)	Retrospective study	Italy	II level NICU (*n* = 45)	48 months (2016–2020)	Integrative model	Pediatrician, nurse and psychologist	From prenatal discussion to postnatal referrals upon request	Prenatal counseling, birthing plan, postnatal care	No	Pregnancy followed and neonatal deaths, the duration of PnPC among all fetuses/neonates	Yes, data shows the presence of a structured service of PPC care that takes care of fetuses and neonates eligible to PnPC and their families, from the time fo diagnosis to bereavement PnPC involvement is growing in regard of neonates with life limiting of life-threatening disease	Small cohort
Tatterton et al., 2022 (Journal of Nursing Scholarship)	Cross-sectional survey, multicentric	UK	30 children’s hospice services providing perinatal palliative care	Electronic survey was sent between May and June 2022	Mixed or collaborative model	N/A	Emotional support, counseling, symptom management, end-of-life care, and bereavement support. They are involved in Advance Care Planning, even before birth	Diagnosis of life-limiting or life-threatening condition antenatally or perinatally	N/A	Describe the role and importance of hospice involvement in PnPC	Yes, increased in perinatal referrals over the years, a family centered biopsychosocial approach to care from antenatal diagnosis (90% accepted antenatal referrals) post-mortem support	Use of qualitative data (i.e., there is no measure of the positive impact on PC on the family); variation of geographies and populations are not representative of all hospices
Parravicini E. et al. 2018 (Journal of Perinatology)	Prospective mixed method self-report study	USA	N/A	N/A	Integrative model	Neonatologist director of the program, nurse clinical care coordinator, social worker, psychologist, speech pathologist, lactation consultant, Child Life specialists and chaplain	N/A	Prenatal counseling, birthing plan, management of postnatal medical care	N/A	Yes	Parental perception concerning the state of comfort maintained in their newborns affected by life-limiting conditions	Questionnaire given at different time intervals after death non-reproducibility of the study: guidelines of a single institution
Currie et al., 2023 (JPSM)	Retrospective study, multicentric	USA	2 NICUs: 1. Children’s of Alabama, level IV NICU (48 beds); 2. Mississipi, Batson Children’s Hospital, Level IV NICU (88 beds). Patients (195)	108 months (2009–2017)	Consultative model	PPC team (not specified)	No standardized protocol or indication to PPC referral. Most PPC consults were initiated for goals of care discussions	Initial PPC consultation occurred a median of 13 days after admission and a median of 17 days before death. Infants with a primary diagnosis of genetic or congenital anomaly received earlier PPC consultation	N/A	Sociodemographic characteristics of NICU infants, pattern of palliative care and intensity during the last 48 h of life	Despite PnPC integration, infants continue to receive high intensity medical care in the last 48 h of life and limited hospice use. Furthermore, racial differences in the intensity of medical care provided at the EoL were found	It was reported the timing of PnPC consultations and not the content or the frequency of communication; risk of documentation bias due to inconsistent or incomplete recording of care elements (such as spiritual support) that may have led to an underestimation of the care provided
Summers et al., 2024 (Arch Dis)	Prospective study	UK	2 NICU (*n* of beds N/A)	60 months (November 2019–2023)	Consultative model children’s hospice-based perinatal palliative clinical nurse specialist (PPCNS) integrated to prenatal round and perinatal services	Nurse specialist and other palliative care specialists (not specified)	Perinatal rounds (pre and post natal, to identify babies potentially benefitting from the referral)	From Prenatal discussion	Yes	Impact of perinatal palliative clinical nurse specialist	-Increase in postnatal referrals -Development of regional perinatal pathway -Increase awareness among professional of services offered by the PPC team -Positive feedback from perinatal teams about the impact of PnPC role	Abstract, few data reported
Nguyen et al., 2018 (Journal of Perinatology)	Observational study with planned sequential interventions (quality improvement project)	USA	Level IV-referral NICU in Philadelphia (n=>200 transported admissions each year) 30-bed unit	24 months (2015–2016)	Consultative mode﻿l	CORE team (Pediatric Palliative Care team) is made up by 2 attendings, a social worker, and nurse practitioner	The survey identified a need for guidelines for consultations, otherwise known as “triggers”	Following the implementation of triggers, the maximum average time to consultation decreased significantly, from 46 to 6 days	Yes	Yes (impact of implementation of triggers for palliative care consultation)	-Increase in PnPC consultation (26% pre vs 46% post) -Increase in level of knowledge of PnPC team role and indication for consultation -Decreased in time until initial consultation -Increase satisfaction among providers	The subjectivity of having one investigator access and interpret the medical record to cross reference with the trigger list
Petteys et al. 2015 (American Journal of Hospice & Palliative Medicine)	Prospective study	USA	Level III NICU with an established pediatric PC program in California (beds n/A)	4 months (August–November 2012f)	Integrative model	1 advanced practice nurse and 1 bachelors prepared Registered Nurse with extensive NICU experience trained in PC with additional medical support available	Physician orders for PC services were based on hospital policy (birth gestational age 28 weeks or less, known/suspected congenital/chromosomal anomalies, and coordination of multidisciplinary support)	N/A	No	Evaluation of PmPC influence of INCU parent stress and satisfaction scores. The Parental Stress or Scale: NICU (PSS: NICU) and Stanford Acute Stress Reaction Questionnaire (SASRQ) measured stress scores at study enrollment, 2 weeks later, and at discharge. Parental satisfaction with care was measured once at discharge. 33 parents were involved: 23 usual care and 10 pnPC	No significant differences in stress scores were found (*P* ¼. 27–1.00). Palliative care parents (100%) were more likely to report being ‘‘extremely satisfied’’ with care than usual-care parents (50%)	Small sample and not randomized design
Engelder et al., 2012 (Advances in Neonatal Care)	Descriptive study	USA	Perinatal hospice program in Southern California	N/A	Collaborative model	Obstetrician, perinatologist, neonatologist, anesthesiologist, specialist/geneticist if appropriate L&D manager, MBU manager, medical/surgical/gynecology unit manager, hospital chaplain, hospital social worker, NICU nurses, hospital case manager, and PPC coordinator	Care is provided from the time of diagnosis through the pregnancy, delivery, and into the period of bereavement. Follow-up to the family continues for 1 year after the death	At the time of diagnosis of a lethal condition	Yes	Impact of Perinatal Comfort Care program implementation	-Increase number of referrals to the program over the years -Family satisfaction with the program -Positive community reaction	N/A
McLaughlin et al., 2020 (Advances in Neonatal Care)	Retrospective medical record review	USA	Level IV NICU (beds N/A). Population = 64 infants	24 months (2015–2016)	Consultative model	The PACT (Pediatric Advanced Palliative Care Team) was composed of 4 physicians, 2 nurse practitioners, and 1 social worker, who provided palliative care services for all inpatients	Assistance with defining goals of care/end-of-life conversations (most frequent), care coordination, symptom management and hospice planning	PACT consultation during hospitalization in NICU/other ICU	No	Comparison of EoL in infants who received PnPC consultation (*n *= 20) and infants who did not (*n* = 44)	Only 20 (31%) infants received PC consultation; however, care sur- rounding end-of-life, between infants who did and did not receive palliative care consultation, was similar. General characteristics between infants who did and did not receive consultation were similar and infants in both groups received invasive procedures	Small sample size, monocentric, Limited to 2 years. It is possible that infants who died outside the NICU and were excluded from the study might have received PnPC consultation

^*^Reason for referral: refractory symptoms management, serious illness, shared-decision making

The studies reviewed showed significant variation in neonatal PnPC models, team composition, and reasons for PnPC involvement, reflecting differences in institutional resources, background, and experience in PnPC and regional healthcare organizations.

### Quality appraisal and risk of bias of the included studies

As detailed in Supplemental Tables 1−4 (Appendix 1), the overall quality of the included studies ranged from low to high. Four cross-sectional studies [21, 22, 28, 31] were assessed as moderate quality, as were the two quasi-experimental studies [29, 32]. Of the four cohort studies evaluated, three [24, 26, 27] were rated as moderate quality, while one [23] was rated as low quality. Additionally, four case series were included, two of which [25, 30] were rated as moderate quality, while the remaining two [19, 20] were considered high quality. Importantly, all the included studies demonstrated congruence between the research methodology and the research questions, as well as consistency in data collection methods, analysis, representation, and the conclusions drawn.

### Palliative care delivery models

The most common models identified were three: consultative, integrative, and team-collaborative approaches, each varying in the degree of integration of palliative care specialists within the neonatal care teams.

**Fig. 2:**
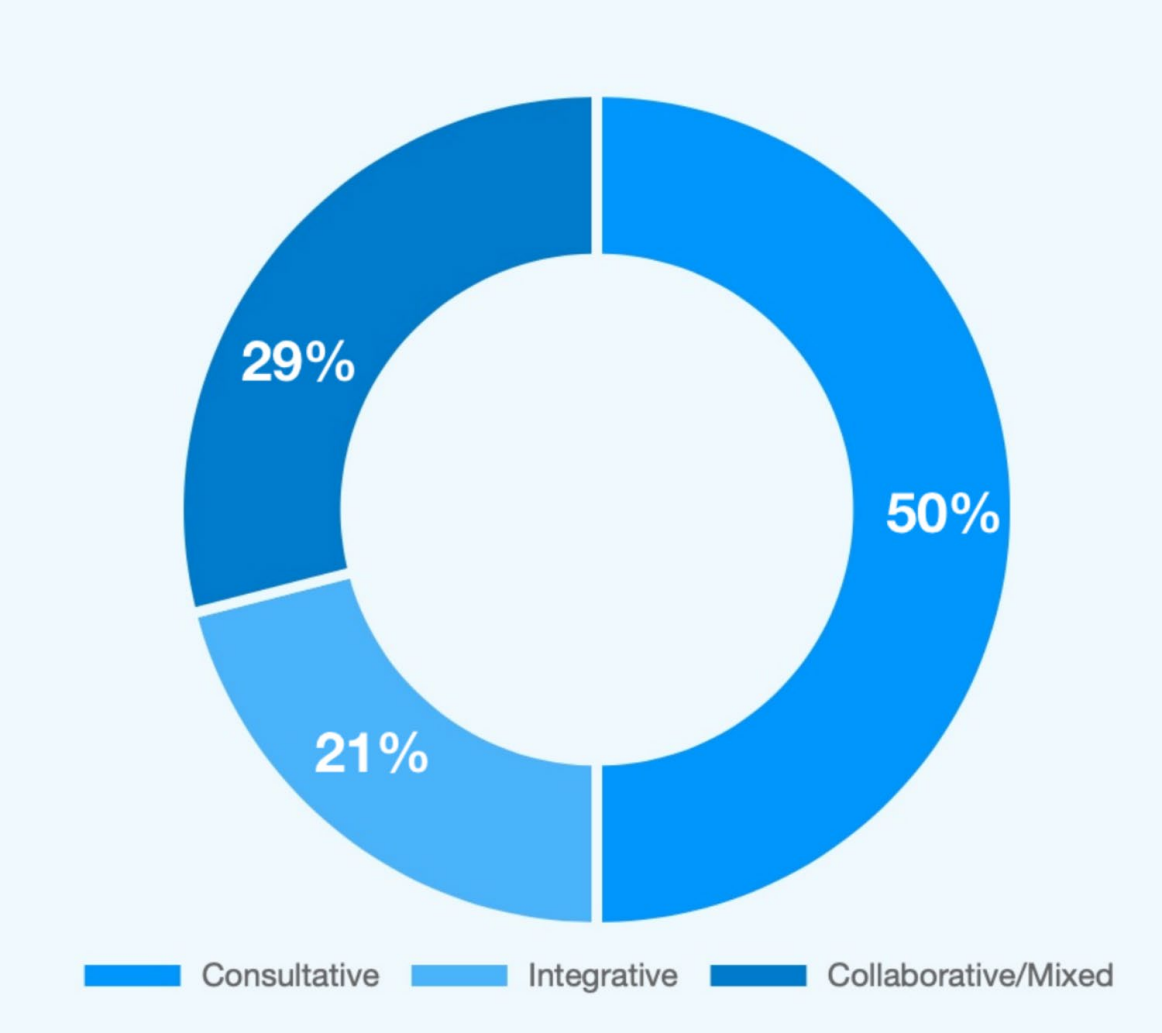
Distribution of perinatal palliative care models in surveyed studies

The consultative model was the most frequent (50%), primarily in high-resource NICUs [19, 21–23, 25, 29, 32] (Fig. 2). In this approach, a specialized multidisciplinary palliative care team is brought in upon referral for specific tasks like goal-setting conversations or end-of-life care. While studies showed this model improved referral rates [19, 23, 32], interprofessional collaboration [19, 23, 29, 32], and provider satisfaction, its main limitations were referral delays [32] and inconsistent integration into daily care [19, 23, 32].Some studies described variations, such as the palliative team taking a central coordinating role for the family [25] or introducing a dedicated palliative care nurse specialist to educate staff and streamline the referral process [23].

On the other hand, Locatelli et al. [29] described the establishment of an innovative PnPC program in a hospital setting in Bologna, Italy, which offers two different models—integrative and consultative—depending on the family’s preference. Moreover, patients could also be referred from outside institutions. Integrative model (3/14, 21% of studies) blended palliative care seamlessly into the standard neonatal care regimen. This is often led by a neonatologist with palliative care expertise who coordinates care continuously from the prenatal period to death or discharge [20',24,28].

A collaborative model was described as an evolution from the consultative approach to fix inconsistencies in end-of-life care [26]. This model emphasizes an early and continuous partnership between the primary neonatal and palliative care teams for shared decision-making and co-management. Engelder’s article [30] was the only one that described in detail how the PnPC service is structured, what services are offered (both outpatient and inpatient care), and the timing of their provision. It was also the only one that addressed the financial aspect and explained how funding was secured to support the program.

**Fig. 3:**
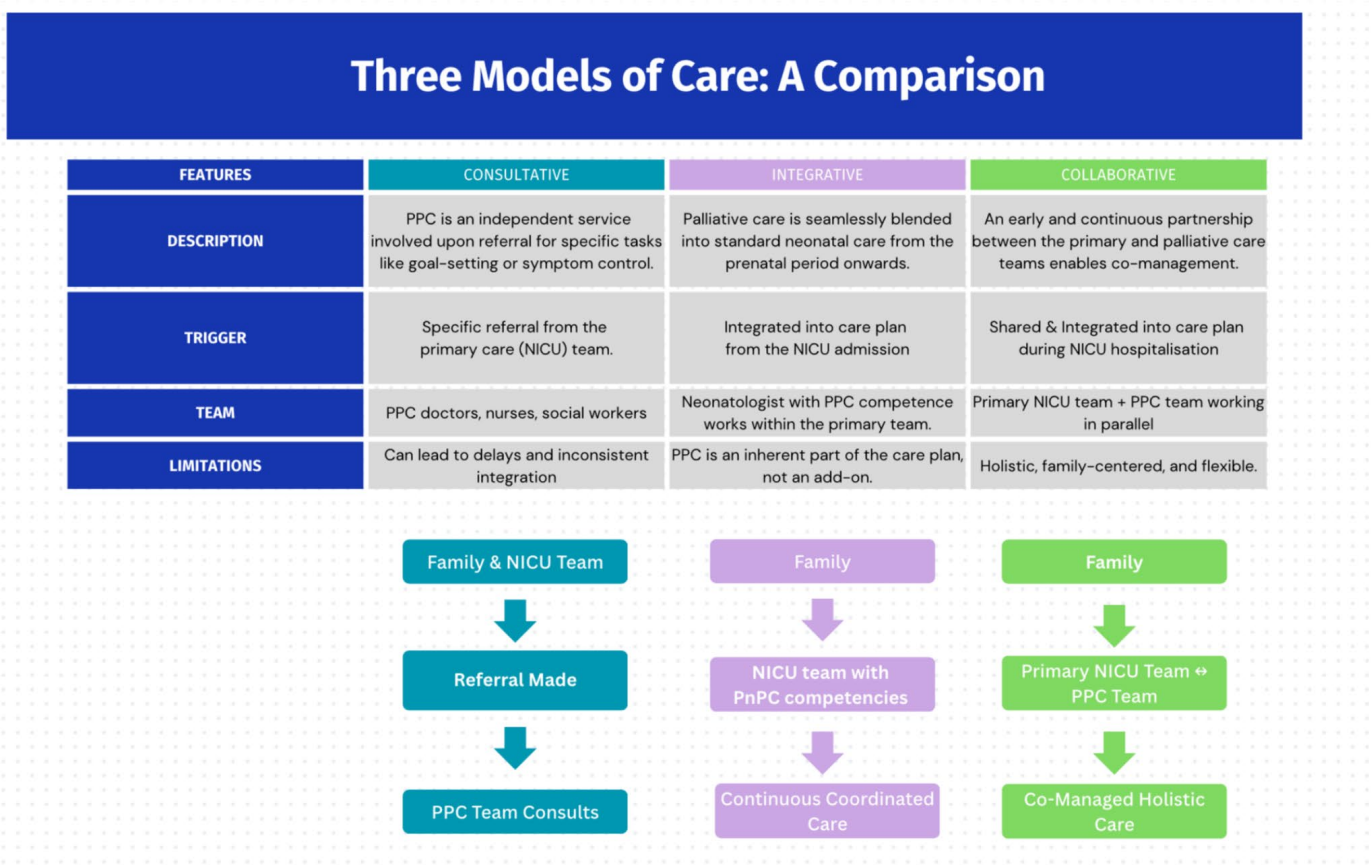
Palliative care delivery models

Tatterton et al. [31] explored the role of UK children’s hospices in PnPC and advance care planning (ACP) through a national survey. Although the article did not explicitly categorize the hospice model as “consultative,” “integrative,” or “mixed,” it described numerous operational features—such as early and continuous involvement, a broad range of services provided from pregnancy onward, and interdisciplinary as well as inter-agency collaboration—that aligned the prevailing model more closely with a collaborative approach. Main characteristics of these three models are summarized in Fig. 3.

### Palliative care team composition

A multidisciplinary approach is essential in PnPC as shown in various studies. Care teams usually include neonatologists for clinical management and palliative care integration, pediatric palliative care specialists for care goals and communication, nurses for continuity of care, daily support and emotional support, and social workers for psychosocial assistance [19, 25, 29]. Psychologists and chaplains may also join to provide mental health and spiritual support within a family-centered care model [28, 32].

Some models involve obstetricians during the antenatal phase, rehabilitation therapists for comfort, and advanced nurse case managers for care planning [23, 29]. Notable examples include a PnPC nursing specialist (PnPCNS) [23] in a children’s hospice that increased referrals and training for healthcare professionals and who had a strategic impact in establishing shared care pathways with perinatal teams, and a Paediatric Advanced Care Team [21] in a NICU that managed clinical and communication tasks effectively. Similarly, Nguyen et al. [32] described the CORE team—comprising two physicians, a social worker, and a specialist nurse—and highlighted how a clearly defined structure and the implementation of “trigger” criteria facilitated earlier and more appropriate consultation requests and enhanced the integration of palliative care within the neonatal setting.

The role of nurses emerged repeatedly across the articles [21–27] in providing continuity of care and contributed to higher parental satisfaction, integrating family preferences into care decisions, and ensuring an approach that was truly person- and relationship-centered.

### Timing of PC involvement

The timing of palliative care involvement varied widely across the studies, though a majority (*n* = 8, 57%) initiated support prenatally upon diagnosis of a life-limiting condition [19, 25–30]. Early integration was linked to better outcomes, including improved parental and health-care providers’ satisfaction [19, 24, 25, 28–32], clearer goals of care [20, 26, 27, 29, 31], reduced aggressive interventions at the EoL [20, 26, 27, 29], and better bereavement outcomes for families [20, 30, 31].

In contrast, some studies reported significant delays [21, 32] with consultations occurring weeks after admission. Notably, Nguyen et al. demonstrated that implementing structured referral triggers—such as checklists and staff education—dramatically reduced the time to consultation from an average of 46 days to just 6 days [32].

### PnPC outcomes and impact

The analysis of different PnPC models shows a positive effect on neonatal end-of-life management and family support. Studies found that a collaborative PnPC model raised care redirection rates significantly (from 35 to 73%) [26] and increased the use of palliative medications before death [26, 27]. Results also showed more family meetings about end-of-life choices [27]. At Children’s Mercy Hospital [19], prenatal PnPC consultations rose from 0.6% in 2011 to 25.3% in 2018, improving care perceptions among providers. Similarly, Bolognani et al. [20] observed that an integrative PnPC model ensured continuity of care from diagnosis through bereavement, with a progressive increase in PnPC consultations over time.

Some studies [20, 21] reported a persistently high intensity of medical care during the last 48 h of life and limited utilization of hospice services; PnPC consultation occurred in only 31% of cases, often too late, with an average age of 91.1 days for consultation. Conversely, early integration of palliative care [32] significantly improved the time to consultation (from 46 to 6 days), improving the timeliness of supportive care. Some studies reported positive parental experiences [24, 25, 28–30] but only one study [28] used a structured standardized questionnaire to score infant comfort.

Regarding guidelines, the integration of PnPC into clinical practice was supported by various educational and organizational strategies across multiple settings [20, 25–30, 32].

Multiple studies described structured programs based on PnPC protocols, including care pathways and staff training [25, 27, 29, 30]. Tewani et al. [25] implemented the PnPC program with ACP in 63% of referred families, while Younge et al. [27] and Parravicini et al. [28] emphasized protocols aimed at achieving neonatal comfort, standardizing birthing plans and comfort care [26–28] or EoL care and a hospice discharge guide [25–29]. In Italy, both Locatelli [29] and Bolognani et al. [20] described structured PnPC integration within regional networks; while only the former explicitly referenced local guidelines, both programs featured defined team roles and standardized processes. Together, these experiences reinforced the central role of formalized guidelines and protocols in delivering coordinated, high-quality PnPC.

## Discussion

This systematic review evaluated palliative care models in NICUs worldwide based on 14 studies, revealing significant heterogeneity in the structure and delivery of PnPC globally and underscoring the dynamic and evolving nature of this crucial field. The majority of included studies were observational, with no interventional trials identified, reflecting the nascent stage of robust evidence generation in this domain. A striking concentration of research from the USA (64%) highlights a geographical imbalance, suggesting a need for more diversified international contributions to understand global variations.

The review identified three predominant PnPC delivery models: consultative, integrative, and team-collaborative approaches. The consultative model remains the most frequently adopted, particularly in high-resource settings. While effective in improving referral rates, interprofessional collaboration, and provider satisfaction [19, 23, 32] it often faces limitations such as delayed referrals and inconsistent integration into daily care. The choice to identify a dedicated professional to PnPC, as described by Summers et al. [23], involving a PnPCNS exemplifies how a consultative framework with dedicated people and a strict collaboration with the NICU clinical setting can facilitate earlier involvement and education. Conversely, the integrative and collaborative models, though less common, represent a more seamless blending of palliative care into routine neonatal care, emphasizing early and continuous partnership from the prenatal period, as demonstrated by Bolognani et al. [20] and Samsel et al. [26]. These models appear to offer comprehensive, co-managed care, addressing the holistic needs of both the infant and family. The transition from a consultative to a collaborative model, as reported by Samsel et al.[26], underscores a recognition of the benefits of early, consistent integration, especially for optimal EoL care.

A critical finding across all models is the multidisciplinary nature of the palliative care team. The pivotal role of nurses, particularly through specialized roles like the PnPCNS [23] and dedicated PnPC nursing teams [24], emerged as central to ensuring continuity of care, enhancing parental satisfaction, and facilitating anticipatory guidance. This multidisciplinary framework is essential for navigating the complex clinical, emotional, and ethical considerations inherent in neonatal palliative care.

**Fig. 4:**
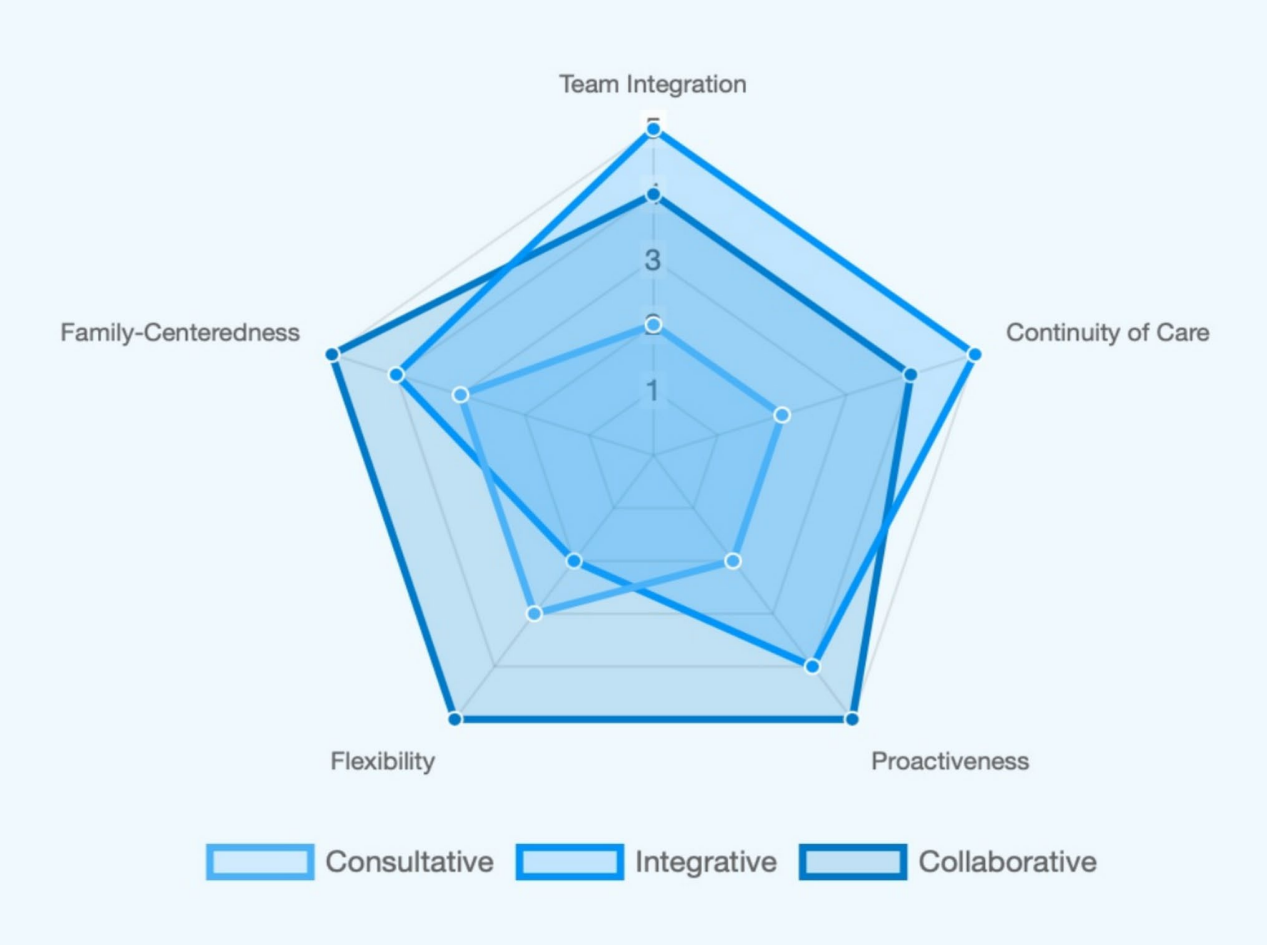
Strengths points of each model are shown in this figure. The five key attributes based on their description are: team integration (how deeply embedded the PPC team is with the primary care team), continuity of care (the seamlessness of care across a patient's journey), proactiveness (how early and automatically care is initiated), flexibility (the ability to adapt the care model to family needs or different situations), family-centeredness (the focus on holistic, biopsychosocial family support)

The timing of palliative care involvement proved to be a critical determinant of outcomes [33, 34]. Early integration consistently correlates with improved parental and healthcare provider satisfaction, clearer goals of care, and reduced aggressive interventions at the EoL [28, 29]. The implementation of standardized antenatal counseling processes and structured trigger criteria for referral, as seen in Nguyen et al.’s work [32], significantly reduced the average time to consultation, improving the timeliness of supportive care. However, despite the progress in the field, several areas for improvement remain. Some studies continue to report a high intensity of medical interventions at the EoL and limited utilization of hospice or specialized palliative services [21, 35, 36], indicating delayed or insufficient integration of dedicated palliative support in certain clinical settings. This variability in the timing and criteria for initiating PnPC activation across different NICUs underscores the need to establish standardized, evidence-based triggers to guide timely referral and activation of PnPC services. In Fig. 4, we compared the theoretical strengths of each model across five key attributes extracted from their descriptions that we summarized over the analysis.

Regarding outcomes and impact on clinical practice, our findings are consistent with existing literature, which indicates a generally positive influence of PnPC models on both the management of neonatal EoL care and the support provided to families [37, 38] but a lack of validated tools to objectively assess the quality and effectiveness of PnPC interventions [39, 40]. Feedback has generally been positive, with studies quantitatively demonstrating high parental perception of infant comfort and overall satisfaction with the care received [24, 28]. However, in many cases, the infant’s comfort was either not directly evaluated or assessed solely in terms of pain, without a broader consideration of multidimensional comfort indicators. The development and application of standardized outcome measures for PnPC remain particularly challenging due to the highly individualized and complex nature of EoL experiences. Furthermore, a significant gap in the literature remains regarding long-term psychosocial outcomes for families following neonatal loss.

Evidence from adult and pediatric non-NICU populations demonstrates that specialized palliative care services correlate with reduced caregiver stress, improved satisfaction, and cost savings [41–44]. These benefits suggest that similar outcomes could potentially be achieved in neonatal settings, and additional research to identify key components that define high-quality, patient- and family-centered care in these sensitive circumstances is needed [40].

Guidelines for neonatal palliative care, such as those proposed by Younge et al. [26], highlighted how structured protocols facilitate earlier integration of palliative services and improve care consistency. Written guidelines and defined consultation criteria are critical given the complex medical and ethical issues in this population and serve as practical tools for multidisciplinary teams. However, regional and institutional variability continues to pose a significant barrier to the standardization of PnPC practices. Differences in healthcare infrastructure, cultural attitudes toward EoL care, and local policies influence how programs are implemented and evaluated [45–47]. Beyond structural and systemic disparities, ethical complexities also contribute to this variability. Decision-making at the EoL for neonates with adverse prognoses is particularly challenging, as determining the best interests of the child involves a nuanced assessment of medical needs, the quality of the child’s relationships with caregivers and family, and the projected quality of life [48]. These multifactorial considerations make ethical deliberations highly individualized and context-sensitive, further complicating efforts to develop universally applicable care standards.

While this review synthesized valuable insights into PnPC models, certain limitations in the included studies warrant consideration. There is still limited knowledge about PnPC as there is a scarcity of information regarding established neonatal palliative care programs and effective interventions. The lack of interventional studies hampers the ability to determine causal links between specific characteristics of PnPC models and patient or family outcomes. Additionally, several studies present incomplete demographic data and are based in high-resource settings thereby limiting the generalizability of their findings to broader, more diverse contexts. The implementation of effective PnPC faces challenges due to insufficient training provided to neonatology fellows, who often lack structured and comprehensive education in EoL care [49, 50]. This gap highlights the need for curricular reforms, as educational initiatives in palliative and EoL care enhance clinical competencies, boost healthcare providers’ confidence, and promote compassionate attitudes [51, 52].

## Conclusion

The reviewed studies highlight the variability in neonatal palliative care models, timing of integration, and team compositions. While the body of evidence supporting the benefits of PnPC continues to grow, substantial barriers remain. These findings suggest that earlier integration of palliative care into neonatal care pathways can enhance support not only for babies and their families, but also for healthcare teams. Future research should prioritize the adoption of rigorous interventional designs, broaden geographic diversity in study populations, and place greater emphasis on evaluating long-term family-centered outcomes, including bereavement support. Integrating practical clinical experiences—such as structured internships and hands-on training—alongside theoretical education, as emphasized by participants in our educational initiatives, is crucial. This approach will not only strengthen the practical competencies of healthcare professionals but also inform the development of evidence-based guidelines and best practice recommendations in neonatal palliative care.

## Supplementary Information

Below is the link to the electronic supplementary material.

ESM 1 DOCX (12.5 KB)

## Data Availability

No datasets were generated or analysed during the current study.

## Abbreviations

ACP: Advance care planning
EoL: End-of-life
NICU(s): Neonatal intensive care unit(s)
PnPC: Perinatal palliative care
PnPCNS: Perinatal palliative care nurse specialist
PRISMA: Preferred Reporting Items for Systematic Reviews and Meta-Analysis

## Declarations

Ethics approval

Not required for this study.
