# Supplementary material for: A systematic review of perinatal palliative care models: challenges and opportunities for the future
Source: Eur J Pediatr. 2025 Oct 12;184(11):678. doi: 10.1007/s00431-025-06459-0 (PMC12515238; doi:10.1007/s00431-025-06459-0)
Supplement: Supplementary file 1 — DOCX (12.5 KB) [file 431_2025_6459_MOESM1_ESM.docx]

| **Criterion** | **Parravicini et al. 2017** | **McLaughlin et al. 2023** | **Currie et al. 2023** | **Tutterton et al. 2022** |
| --- | --- | --- | --- | --- |
| **1.Were the criteria for inclusion in the sample clearly defined?** | + | + | + | + |
| **2. Were the study subjects and the setting described in detail?** | + | + | + | + |
| **3.Was the exposure measured in a valid and reliable way?** | + | + | + | + |
| **4.Were objective, standard criteria used for measurement of the condition?** | + | + | + | + |
| **5.Were confounding factors identified?** | - | - | N/A | - |
| **6.Were strategies to deal with confounding factors stated?** | - | - | - | - |
| **7.Were the outcomes measured in a valid and reliable way?** | + | + | + | - |
| **8.Was appropriate statistical analysis used?** | + | + | + | + |
| **Quality of the study** | MODERATE QUALITY | MODERATE QUALITY | MODERATE QUALITY | MODERATE QUALITY |

**Table 1.** Methodological quality of the Cross Sectional Studies

| **Criterion** | **Locatelli et al.**  **2020** | **Nguyen1 et al.**  **2018** |
| --- | --- | --- |
| **1.Is it clear in the study what is the “cause” and what is the “effect” (i.e. there is no confusion about which variable comes first)?** | + | + |
| **2. Was there a control group?** | - | - |
| **3. Were participants included in any comparisons similar?** | + | + |
| **4. Were the participants included in any comparisons receiving similar treatment/care, other than the exposure or intervention of interest?** | + | + |
| **5. Were there multiple measurements of the outcome, both pre and post the intervention/exposure?** | + | + |
| **6. Were the outcomes of participants included in any comparisons measured in the same way?** | + | + |
| **7.Were outcomes measured in a reliable way?** | N/A | + |
| **8. Was follow-up complete and if not, were differences between groups in terms of their follow-up adequately described and analyzed?** | N/A | N/A |
| **9.Was appropriate statistical analysis used?** | N/A | - |
| **Quality of the study** | MODERATE QUALITY | MODERATE QUALITY |

**Table 2.** Methodological quality of the Quasi experimental studies

| **Criterion** | **Samsel et al.2015** | **Younge et al. 2015** | **Petteys et al.**  **2015** | **Summer et al 2022** |
| --- | --- | --- | --- | --- |
| **1. Were the two groups similar and recruited from the same population?** | + | + | + | + |
| **2. Were the exposures measured similarly to assign people to both exposed and unexposed groups ?** | + | + | + | + |
| **3. Was the exposure measured in a valid and reliable way ?** | + | + | + | + |
| **4. Were confounding factors identified?** | + | + | N/A | N/A |
| **5. Were strategies to deal with confounding factors stated?** | - | - | - | - |
| **6. Were the groups/participants free of the outcome at the start of the study (or at the moment of exposure)?** | N/A | N/A | + | + |
| **7. Were the outcomes measured in a valid and reliable way ?** | + | + | + | - |
| **8. Was the follow up time reported and sufficient to be long enough for outcomes to occur?** | N/A | N/A | + | N/A |
| **9. Was follow up complete, and if not, were the reasons to loss to follow up described and explored?** | N/A | N/A | N/A | - |
| **10.Were strategies to address incomplete follow up utilized?** | N/A | N/A | N/A | N/A |
| **11. Was appropriate statistical analysis used?** | + | + | + | - |
| **Quality of the study** | MODERATE QUALITY | MODERATE QUALITY | MODERATE QUALITY | LOW QUALITY |

**Table 3**. Methodological quality of the Cohort Studies

| **Criterion** | **Tewani et al. 2002** | **Tucker et al.**  **2020** | **Engelder et al. 2012** | **Bolognani et al. 2020** |
| --- | --- | --- | --- | --- |
| **1.Were there clear criteria for inclusion in the case series?** | + | + | + | + |
| **2. Was the condition measured in a standard, reliable way for all participants included in the case series?** | + | + | + | + |
| **3.Were valid methods used for identification of the condition for all participants included in the case series?** | + | + | + | + |
| **4. Did the case series have consecutive inclusion of participants?** | N/A | + | N/A | N/A |
| **5.Did the case series have complete inclusion of participants?** | N/A | + | N/A | + |
| **6. Was there clear reporting of the demographics of the participants in the study?** | - | + | - | + |
| **7.Was there clear reporting of clinical information of the participants?** | + | + | - | + |
| **8. Were the outcomes or follow up results of cases clearly reported?** | + | + | + | + |
| **9.Was there clear reporting of the presenting site(s)/clinic(s) demographic information?** | + | + | + | + |
| **10.Was statistical analysis appropriate?** | N/A | + | N/A | + |
| **Quality of the study** | MODERATE QUALITY | HIGH QUALITY | MODERATE QUALITY | HIGH QUALITY |

**Table 4.** Methodological quality of the Case Series
